# Centricity on patients using healthcare reputation management strategies to improve dentistry image in Romania

**DOI:** 10.22336/rjo.2023.59

**Published:** 2023

**Authors:** Cristina Stanciu (Neculau), Aida Geamănu, Roxana Monica Purcărea, Alin Gabriel Sterian

**Affiliations:** * Department of Marketing and Medical Technology, “Carol Davila” University of Medicine and Pharmacy, Bucharest, Romania; ** Department of Ophthalmology, “Carol Davila” University of Medicine and Pharmacy, Bucharest, Romania; *** “Dr. Carol Davila” Clinical Hospital of Nephrology, Bucharest, Romania; **** Department of Pediatric Surgery and Orthopedics, “Carol Davila” University of Medicine and Pharmacy, Bucharest, Romania; “Grigore Alexandrescu” Emergency Hospital for Children, Bucharest, Romania

**Keywords:** reputation management, online image, healthcare, medical reputation, quantitative research

## Abstract

Reputation is seen as an abstract concept that focuses on patients’ perceptions of a particular medical institution. A good image of these practices among patients makes them return to them and also helps attract new patients and increase their reputation and revenue. The paper aimed to explore the respondents’ perceptions of the way the image of online dental practices can influence their reputation management. For this purpose, a quantitative study was conducted on a sample of 107 respondents. Data collection was carried out using a questionnaire that was posted on an online platform. Data analysis was done by using IBM SPSS software. The results emphasized that patients think that the image of dental practices in the online environment can influence their reputation to a high extent.

## Introduction

*The importance of reputation management in healthcare institutions*

Reputation is directly influenced by patient experience and fluctuates according to patient satisfaction with the services provided [**1**]. Reputation is built up over time and is very easy to damage or lose [**2**]. A widely accepted definition of this concept belongs to Fombrun and Shanley [**3**], according to whom reputation consists of a representation of an organization’s past actions and prospects comparable to those of other competing businesses. A good reputation attracts investors and low capital costs, thus improving competitiveness [**4**].

Trust is crucial in choosing a specialized institution for both minor and major interventions, leading patients to prefer institutions with a better reputation when they have to choose between several organizations offering similar services, as they consider that reputation is especially important when their health is at stake [**5**].

With the development of the medical system and new specialized services, more and more hospitals and private clinics have emerged. Thus, patients do not have to be strictly oriented toward a certain clinic when they wish to have a medical investigation or an intervention, which turns their choice into something based on the organizational reputation or the quality of the medical act. Some patients choose a particular doctor, even if the reputation of that clinic is not satisfactory enough for them [**6**].

According to Pilny and Mennicken [**7**], patients who choose a medical institution at a greater distance from their location are more attentive to its reputation, as opposed to those who choose a clinic or hospital that is closer to their home or location. They also found that there is a connection between the reputation of the medical institution and the degree of difficulty of the medical interventions carried out in that place. The more difficult a procedure is, or the fewer institutions have the capacity and equipment to perform it, the more patients value the clinic or hospital, which automatically increases the institution's reputation. The reputation of a healthcare organization also has an important impact on its financial situation, as a high reputation will influence the number of investors, which will automatically increase the number of patients [**8**].

Reputation is also closely related to patients’ perceptions of the quality of healthcare services provided by healthcare institutions and the degree of respect for patients’ rights, satisfaction, and management of quality practices [**9**]. The reputation of a healthcare institution can easily be affected by a single negative incident. Thus, even the highest-rated clinics and hospitals are vigilant to reputation-based rankings by patients [**10**]. Negative patient ratings cause the loss of current patients, and at the same time discourage new patients from seeking the medical services of that institution. A poor reputation of a healthcare institution is very difficult to restore and requires time but also a strong strategy aimed at regaining lost patient trust.

*The reputation of medical institutions during the Covid 19 pandemic*

The healthcare field played a crucial role in the COVID-19 pandemic that influenced the world in 2020, which has raised a high degree of admiration for healthcare professionals. The SARS-CoV-2 virus emerged in 2020 in Wuhan and caused several ailments in the human body, some of which even led to deaths [**11**]. The whole globe has been affected by the pandemic crisis that has led to the collapse of healthcare systems in several European countries and also to the collapse of economies [**12**]. Due to the restrictions imposed because of the pandemic, the process of digitization has gained influence and most of the physical processes have been transferred online. The healthcare system was no exception to this digitization process, with many virtual clinics and various e-health services emerging due to the pandemic crisis, enabling patients to be consulted remotely and avoiding the need to travel to specialized medical centers.

Today, thanks to Web 2.0 technologies, patients can join online communities where they can get both the necessary information on the quality of care and recommendations from medical specialists based on other users’ experiences. The benefits of these communities have been studied from both patient and physician perspectives [**13**,**14**].

*Online reputation of healthcare institutions*

The online reputation of healthcare institutions is very important, even if sometimes overlooked. An important aspect to stress is that it matters a lot to both patients and investors. Online reputation can be established with a published questionnaire that is transmitted via research platforms. Patients have the opportunity to see that their opinion matters, and they can help to some extent in creating the online reputation of a medical institution. If a particular specialized institution does not have an online reputation or has one but it is quite poor, then investors and patients cannot confide, invest in, or use the medical services it offers. Moreover, search engines are used to rank the websites of medical institutions according to their online reputation. Typically, healthcare organizations that are listed on the first page are the most visited by users. The emergence of blogs and social networks in the Web 2.0 era has enabled the sharing of medical experiences through specialty websites such as RateMDs which collects information related to patient experience and satisfaction in the provision of medical care and allows the evaluation of specialists [**15**,**16**]. Through specialty websites, doctors can create an online reputation, which is widely based on feedback received from patients [**17**].

Ongoing technological progress has led to the creating of a computational infrastructure for e-health. E-health or electronic health refers to using IT&C technology to store and transfer health information [**18**]. According to Dowling [**19**], an organization’s reputation is an intangible asset that depends on the actions of that organization as an entity but also on how it communicates within the market. Globalization of business can affect the reputation of the business and the theoretical context includes the literature on the effect of the origin of that country and the impact of the original business [**20**,**21**]. The term “reputation” plays a key role in the process of building trust between the organization and patients [**22**,**23**].

As stated above, the reputation of a healthcare institution is probably its most fragile intangible asset. The market has changed with the introduction of the Internet into the daily lives of individuals [**24**]. Through social media, individuals can communicate directly with organizations, which was helpful during the COVID-19 pandemic as it reduced or in some cases even eliminated the need to travel to physical institutions [**25**]. Nowadays, anyone can create a blog or website, where they can share their personal experiences of the services offered by particular institutions [**26**]. The digital market is growing, and traditional media markets have become passé [**27**]. With the emergence of social media platforms, the communication strategy of healthcare institutions has also changed. The second half of the 21st century has seen massive technological progress, and this has been characterized by accelerating digitalization, with most businesses moving much of their identity online [**28**,**29**].

## Materials and methods

Based on the importance of the reputation of dental practices in the online environment, we considered it necessary to carry out quantitative research aimed at identifying the importance of the image of these practices in terms of reputation management. This study was undertaken in this field because it was observed that the health of the teeth can influence the health of other organs, such as that of the eyes. Very often, the dental infections can affect the vision of patients.

This study aimed to identify respondents’ perceptions of how the image that dental practices have in the online environment can influence their reputation. The quantitative research was conducted from July 1, 2023, to August 1, 2023, on 107 respondents. Data was collected by using a questionnaire that was posted on an online platform and subsequently distributed to respondents. The questions in the questionnaire were designed to identify the respondents’ opinions on how the image that dental practices have in the online environment can influence reputation management.

## Results

A primary objective of this paper was to identify the main means used by respondents to gather information about the dental practices they are interested in. The analysis showed that a large proportion of respondents (61.7%) collect this information online, while 32.7% of respondents indicated that they collect this information from both online and traditional sources. Only a small proportion of respondents (5.6%) indicated that they get their information from both online and offline sources.

Regarding the demographic profile of the respondents (**Table 1**), it should be noted that most of the respondents who participated in the survey (73.8%) were between 26-35 years old, 21.5% of them were between 36-45 years old, while only 4.7% of them were between 18-25 years old. Regarding the gender distribution of the respondents, it was observed that 58.9% of them were women, while 41.1% of them were men. As for the distribution according to the respondents’ area of residence, 68.2% of them lived in urban areas, while the rest (31.8%) lived in rural areas.

**Table 1:** Respondent profile

Category		Frequency	Percentage (%)
Environment from which respondents gather their information on dental practices	Online environment	66	61.7
	Traditional environment	6	5.6
	From both online and traditional environments	35	32.7
Age	18-25 years old	5	4.7
	26-35 years old	79	73.8
	36-45 years old	23	21.5
Gender	Women	63	58.9
	Men	44	41.1
Residence environment	Rural	34	31.8
	Urban	73	68.2

Another aspect that has been studied in this paper concerns the main sources of information used by respondents to inform themselves about the dental health services offered in certain practices. Most respondents (33.6%) indicated that they got their information from websites. They accessed the websites of dental practices or partners to find out about the services they offer, the doctors who work there, and the prices they charge. 28% of respondents stated they found out about them on social networks. They accessed the pages of dental practices on social media to find updated information on the services offered, the prices charged, or possibly the offers available at their level. 24% of the respondents indicated that they got their information from forums, where people discuss various dental practices that specialize in specific problems, while 14% of them indicated that they got their information from blogs (**Table 2**).

**Table 2:** Sources of information that respondents use to inform themselves about the services offered in dental surgeries

Sources of information	Frequency	Percentage (%)
Web sites	36	33.6
Forums	26	24.3
Blogs	15	14
Social networks	30	28

Another objective of this research was to identify the main elements of the online environment that respondents consider when choosing a dental practice. It should be noted that most of them (42.1%) considered the opinion that other patients had about the practice (**Table 3**). This showed that they were more interested in the reviews received from patients who had previously visited the dental clinic and who expressed their satisfaction afterward. On the other hand, 32.7% of the participants indicated that they considered the opinion that other patients had of the doctors who worked in dental surgeries. It can be observed that feedback was important for patients both about the practice (e.g., facilities, location, cleanliness, etc.) and about the doctors working there (e.g., their experience, quality of service, procedures, etc.). 15% of the respondents mentioned that the rating of the dental practice by other patients was of high importance to them, as they believed that it illustrates the satisfaction they felt after using the services of the dental practice. A small proportion of respondents showed that the number of people who indicated that they had visited a dental surgery was important to them (10.3%), as they felt that the more a dental surgery was in demand, the higher its reputation, which illustrated that the services provided there were of a higher quality.

**Table 3:** The main aspects of the online environment that respondents consider when choosing a dental practice

Online elements that respondents consider when choosing a dental practice	Frequency	Percentage (%)
Other patients’ opinions about the clinic	45	42.1
What other patients think about the doctors in the clinic	35	32.7
Number of people who mentioned that they had been in the office	11	10.3
Score obtained from reviews received	16	15

Another view that has been studied in this research concerns the main aspects that patients consider when choosing a particular dental practice (**Table 4**). It could be observed that 76.6% of them considered the image that the dentist had online, while 23.4% of them considered the image that the dental practice had online. This showed a much higher proportion of people for whom the doctor’s image was important, as they believed that the doctor was the one who provided the medical service and for this reason, he needed to have an image as good as possible. 

**Table 4:** Aspects that respondents consider when choosing a dental surgery

Key issues	Frequency	Percentage (%)
The online image of the dentist’s image	82	76.6
The online image of the medical practice	25	23.4

Another objective of this research was to identify the respondents’ opinions on the importance of the online image of dental practices to improve their reputation. Following the interpretation of the data, it was observed that the respondents considered that the image of dental practices in the online environment is more important (average obtained - 3.96), as it has the role of permanently increasing the reputation of dental practices. Thus, the better the image of dental practices, the higher their reputation among patients.

In terms of respondents’ perceptions of the main actions that dental practice managers should take to improve their online reputation, it was noted that 32.7% of respondents felt that they should constantly update their website and post several reviews from patients they had on social media. 25.2% of respondents stated that dental practice managers should encourage their existing patients to offer them feedback online so that potential visitors could make an idea about their opinion. Only 9.3% of those surveyed stated that these organizations should implement a range of mobile apps to make it easier for them to communicate with patients (**Table 5**). 

**Table 5:** Actions that dental practices should take online to increase their reputation

Actions	Frequency	Percentage (%)
Constant updating of the website	35	32.7
Receiving reviews on social networks	35	32.7
Encouraging patients to provide an assessment in the online environment	27	25.2
Implementation of mobile applications to facilitate online communication with patients	10	9.3

## Discussion

At the level of a health care institution, reputation has the role of integrating expectations about future behavior at the level of an organization and shaping the image of an institution either by its members or by external factors [**30**]. Reputation is based on past actions and organizational behavior but also patient perception. Each experience with practice results in a better or worse reputation [**31**]. A poor reputation will negatively influence the practice’s work and considerably reduce its competitiveness in the market. In medical educational institutions, reputation is seen as a sum of impressions of key stakeholders in the communication process [**32**]. On the other hand, in medical practices, it takes time to build a positive reputation, which implies an institutional commitment to achieving excellence in the field of practice [**33**].

Previous studies in this field have shown that the reputation of a dental practice is one of the key factors influencing both patient and practitioner performance [**34**]. The literature emphasizes that a high reputation of such an institution can indicate a high quality of care [**35**]. Trust in the medical system is defined by risk and uncertainty and plays an important role in the conduct of medical care and the adoption of new digital services [**36**]. The degree of trust in the medical services provided in a dental practice indirectly influences the quality of medical care based on patient satisfaction and patient relationships with physicians. The level of trust refers to patients’ opinion of dentists [**37**]. Trust can be considered an indicator of the quality of medical care through patient experience [**38**].

In the field of dentistry, reputation is of increasing importance. Patients use the services of dentists for both minor procedures (e.g., cavity removal) and more complex procedures (e.g., extractions), and because of this, they often make the decision to go to a particular practice or doctor based on their reputation or the trust they have in them. In terms of patient confidence in this field of practice, it should be noted that patients feel safer when they have information about the dentist, his reputation, and his practice. They are often reluctant to use certain medical services due to a lack of information about the quality of the medical care [**39**].

The COVID-19 pandemic has greatly affected the reputation of these practices. Thus, some of them had to change their strategy to cope with the turbulence in the external environment. They turned to new means and communication tools to strengthen or enhance their reputation at that time.

Following the COVID-19 pandemic, an increased need for the use of strategic public relations in this sector has been observed. The response of healthcare organizations was to implement a range of public policies to help develop and maintain a positive reputation [**40**]. Several research studies in this field have found that the discipline of public relations is designed to focus on trust and reputation management for these institutions [**41**]. Reputation management and the development of long-term relationships with stakeholders are two key features of public relations, and effective reputation management requires good communication with the public as well as adherence to ethical standards in practice [**42**].

This paper aimed to identify patients’ opinions on the importance of dental practices’ image in the online environment to reputation management. We chose to conduct this research in the dental field because the health of the teeth can influence the health of the other organs in the body. Thus, over time, a close connection has been observed between the health of the teeth and the eyes. Studies have shown that dental infections can gradually lead to the appearance of ophthalmia, defective vision, orbital cellulitis, and blindness.

The analysis of the data showed that a large proportion of those who participated in the study gathered their information about dental practices from the online environment (61.7%), they mainly turned to specialist websites (33.6%) and information posted on social networks (28%).

The research revealed that when respondents look for a particular dental practice online, they mostly consider the opinion of other patients about the practice (42.1%), the opinion of other individuals about the doctors working there (32.7%), and the rating of the dental practice by other patients (15%). The results showed that respondents believe that the online image of dental practices can influence their reputation. In addition, to increase the reputation of dental practices online, dental practice managers should give more importance to the information that is posted on their websites (32.7%), encourage current patients to offer feedback on social networks (32.7%) and also on other online tools (25.2%). 

Given the results that have been obtained in this study, further research must be conducted in the future to emphasize the main factors in the online environment that can influence reputation management. In addition, the main actions that should be carried out at the level of each online communication environment by the management of dental practices to permanently improve their reputation should be determined.

## Conclusion

Reputation is an important characteristic in the medical system, as the number of patients and the proper functioning of dental practices depend on it. When stakeholders trust the medical services offered in certain dental practices, they will recommend them either through direct or online means, which will increase their reputation in the market. The opposite situation can also occur when the medical services do not live up to patients’ expectations, which automatically leads to a low reputation among these dental practices. Previous studies have shown that the reputation of such a medical institution is closely linked to the perception patients have of it. Thus, how patients are treated by the medical staff significantly influences their perception of the quality of the services provided in dental surgery.

When considering the reputation of a dental practice, their managers should also study patients’ perceptions of the quality of care. This should be assessed in terms of the degree of satisfaction with the medical services provided. This is important because these aspects will go a long way toward establishing a good or bad reputation for the institution. Patients form an overall picture of a dental practice based on the evaluation of past personal experiences, the attitude of the medical staff, or, in some cases, the experience of other patients shared either directly or through social media or websites dedicated to the evaluation of these practices.

**Conflict of Interest Statement**

The authors declare no conflict of interest.

**Informed Consent and Human and Animal Rights Statement**

Written informed consent was obtained from the participants.

**Authorization for the use of human subjects**

Ethical approval: The research related to human use complied with all relevant national regulations and institutional policies, followed the tenets of the Helsinki Declaration, and was approved by the Ethics Committee of OnDental - Microscope dentistry (code 7/23.07.2023).

**Acknowledgments**

Publication of this paper was supported by “Carol Davila” University of Medicine and Pharmacy, Bucharest, Romania, through the institutional program Publish not Perish. 

**Sources of Funding**

This study was funded by “Carol Davila” University of Medicine and Pharmacy, Bucharest, Romania, through the institutional program Publish not Perish.

**Disclosures**

None.

**Data availability**

Not applicable.
